# Supplementary material for: A whole-animal phenotypic drug screen identifies suppressors of atherogenic lipoproteins
Source: eLife. 2026 Aug 27;14:RP105314. doi: 10.7554/eLife.105314 (PMC13521566; doi:10.7554/eLife.105314)
Supplement: Supplementary file 2 [file elife-105314-code1.zip › JHDL_primary_result/ENOXOLONE (18beta-glycyrrhetinic acid)/ENOXOLONE (18beta-glycyrrhetinic acid)_JHDL_5_009_009_2019_05_17_11_08.pdf]

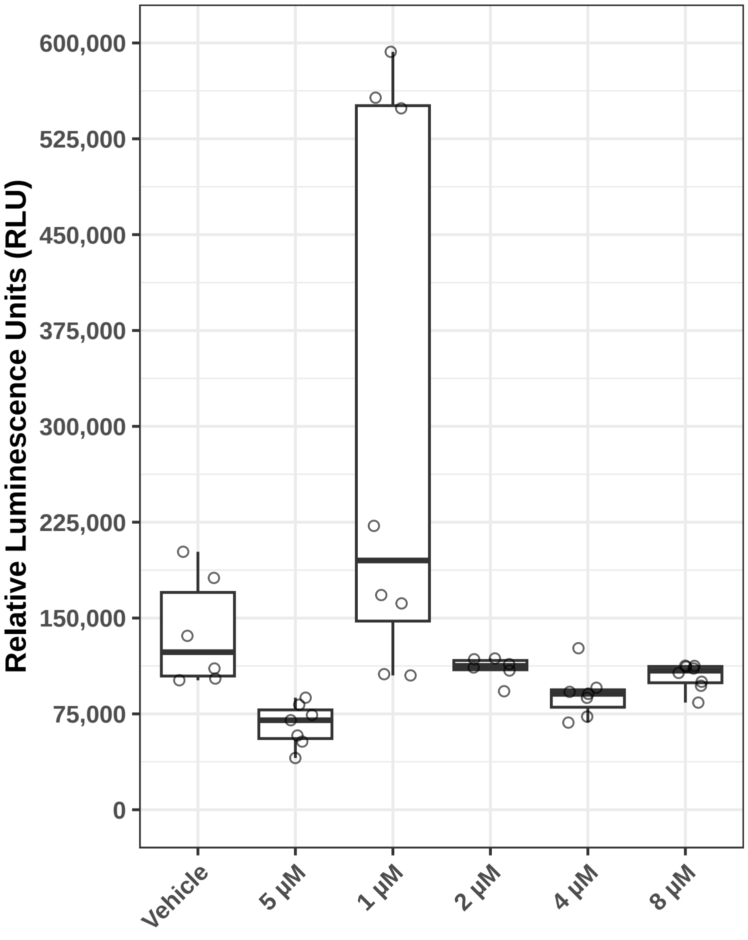
Relative Luminescence Units (RLU)
600,000
525,000
450,000
375,000
300,000
225,000
150,000
75,000
0
Vehicle
5 µM
1 µM
2 µM
4 µM
8 µM
